# Electrochemical Assessment of *Rhus typhina* L. Leaf Extract as a Novel Green Corrosion Inhibitor for OL37 in 1 M HCl Medium

**DOI:** 10.3390/molecules30122660

**Published:** 2025-06-19

**Authors:** Denisa-Ioana Răuță (Gheorghe), Florina Brânzoi, Roxana-Doina Truşcă, Sorin-Marius Avramescu, Ecaterina Matei

**Affiliations:** 1 Biotechnical Systems Engineering Doctoral School, National University of Science and Technology Politehnica Bucharest, 060042 Bucharest, Romania; deni.gheorghe@yahoo.com; 2 Institute of Physical Chemistry-Ilie Murgulescu, 202 Splaiul Independentei, 060021 Bucharest, Romania; fbrinzoi@chimfiz.icf.ro; 3 Faculty of Applied Chemistry and Material Science, National University of Science and Technology Politehnica Bucharest, Splaiul Independentei 313, 060042 Bucharest, Romania; truscaroxana@yahoo.com; 4 Faculty of Animal Productions Engineering and Management, University of Agronomic, Sciences and Veterinary Medicine of Bucharest, 011464 Bucharest, Romania; 5 Faculty of Materials Sciences and Engineering, National University of Science and Technology Politehnica Bucharest, 060042 Bucharest, Romania

**Keywords:** corrosion inhibitors, plant extract, *Rhus typhina* L., OL37, HPLC, LC-MS, electrochemical methods

## Abstract

This study evaluates the corrosion-inhibiting effects of the methanolic (P_1_) and the hydroalcoholic (P_2_) extracts of the *Rhus typhina* L. leaves on carbon steel (OL37) in 1 M HCl. Extracts were prepared with microwave-assisted extraction and characterized using HPLC and LC-MS. Electrochemical methods (OCP, EIS, PDP) and surface analyses (SEM, EDX) assessed the performance of both extracts. The results showed that the P_1_ and P_2_ extracts significantly reduced corrosion rates by forming protective layers on the metal surface, with inhibition efficiencies exceeding 90%, at 1000 ppm concentration, for P_1_ (93%), for P_2_ at 800 ppm (91%) and 1000 ppm (94%). The P_2_ extract demonstrated superior long-term performance, maintaining protection after 96 h of immersion. The extracts function as mixed-type inhibitors, affecting both anodic and cathodic reactions, with physicochemical adsorption demonstrated by the Langmuir isotherm. Overall, the *Rhus typhina* leaf extracts, particularly the P_2_ extract, offer a promising, eco-friendly approach to corrosion prevention in acidic environments.

## 1. Introduction

**Figure 1:**
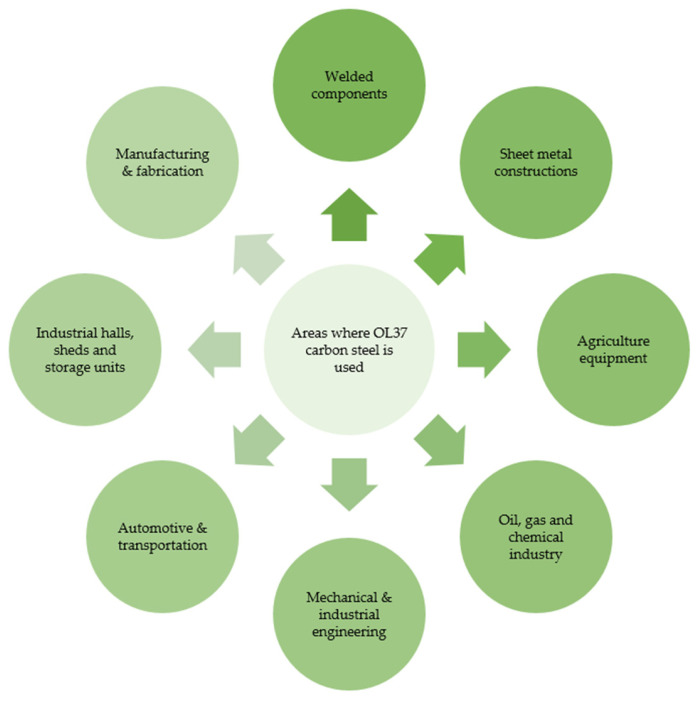
Diagram representation of the industries that use carbon steel.

The uses of carbon steels are numerous, and include kitchen appliances and structural elements. Considering its mechanical qualities and affordability, carbon steel is the material chosen for pipeline construction, and is widely utilized in the oil and gas sector [1]. Because carbon steel is vulnerable to corrode during industrial processes (Figure 1), substantial levels of corrosion inhibition are required for operations to be both safe and economical [2] Corrosion is defined as the degradation of the metallic materials caused by adverse interactions with the surrounding environment, leading to the loss of their physical and chemical properties [3,4,5,6]. Corrosion leads to global economic losses ranging from USD 700 billion to USD 1 trillion annually, and poses serious safety risks due to structural degradation [7,8]. In many industries, such as oil and gas, construction, marine, aircraft, petrochemical, military, and ceramics, corrosion is a major concern.

Corrosion rates are especially high in acidic conditions in fields that rely extensively on mild steel infrastructure, resulting in yearly losses estimated to be billions of dollars (USD). Preventing acid–metal contact is therefore the highest priority. By minimizing the direct interaction between metals and acids, organic inhibitors, when they are applied, create a barrier that prevents corrosion [9,10]. An inhibitor is a chemical or natural material that can reduce or stop corrosion in corrosive environments when added in a limited quantity (1 to 15,000 ppm) [11]. An inhibitor can reduce corrosion by affecting the components of a corrosion cell, causing anodic and cathodic resistance, and diffusion inhibition [12].

Corrosion inhibitors are widely used for their simplicity, cost-effectiveness, adaptability, and superior performance in reducing corrosion across diverse environments [13,14]. Currently, various types of corrosion inhibitors have proven effective in preventing corrosion under different conditions, providing significant economic benefits [15]. Conventional inhibitors (imidazolines, mercury salts, phosphates, and polyphosphates) are costly and toxic for the environment, whereas natural alternatives are more environmentally friendly and cost-effective.

Green chemistry is gaining interest for environmental protection and human health [13]. Until now, considerably research has been invested into designing scalable green corrosion inhibitors, including ionic liquids [16,17,18], natural polymers [19,20,21], plant extracts [1,22,23,24], and amino acids [25,26,27].

Plant extracts, rich in flavonoids, alkaloids, triterpenes, sterols, and fatty acids, are favored as green corrosion inhibitors due to their abundant sources and lower environmental impact. They are extensively utilized in the machinery, chemical, energy, oil and gas extraction, and refining industries [13]. Plant-derived alternatives tend to perform best at higher concentrations [15]. Plant extracts used as corrosion inhibitors offer numerous advantages, such as availability, affordability, non-toxicity, and biodegradability. These green inhibitors present a sustainable and cost-effective alternative to conventional methods, primarily due to their low toxicity and environmentally friendly nature [28,29]. The use of plant extracts as corrosion inhibitors dates back to 1930, when *Chelidonium majus* and other species were employed in pickling baths [30]. More recently, Eddy et al. [31] emphasized the application of vegetable and fruit extracts as effective green inhibitors, particularly under acidic conditions.

Various plant extracts have been discovered to be just as useful as classical corrosion inhibitors, and can be utilized as green corrosion inhibitors. They have a lot of active adsorption centers [32], which can effectively prevent corrosion and build a protective film with the metal surface [15,33]. The effectiveness of using these organic inhibitors to prevent corrosion in carbon steel has been acknowledged in a number of published studies. A considerable number of these studies have investigated the inhibition mechanism and efficiency of organic inhibitors in a carbon steel, 1 M HCl solution system [34]. Numerous scientific studies have investigated the corrosion-inhibiting effect of several natural plant extracts [35,36,37,38,39,40,41,42,43,44,45] as corrosion inhibitors. The most recent examples of various plant part extracts acting as green corrosion inhibitors on different metal surfaces and in diverse corrosive environments are shown in Table 1.

*Rhus typhina* L. (staghorn sumac), a species native to Eastern North America, is known for its wide range of pharmacological properties attributed to various parts of the plant [55,56]. The leaves have been reported to possess antioxidative, anticarcinogenic, antiviral, and antimicrobial properties [57]. The fruit has been reported to show significant biological potential, including antioxidative, antimicrobial, anti-inflammatory, and cytotoxic effects against selected cancer cell lines [58]. The wood has been reported to exhibit both antioxidative and antitumorigenic effects [59]. Phytochemical analyses of the *Rhus typhina* extracts have identified a rich profile of bioactive compounds, including phenolic acids (gallic acid and tannic acid are notably present in the leaves and bark) [60], flavonoids (compounds such as quercetin, kaempferol, and myricetin have been detected in the leaves) [61], hydrolyzable tannins, and organic acids (the fruit contains organic acids, contributing to their astringent and acidic taste, as well as malic, citric, and tartaric acids) [55,62].

The chemical composition of the *Rhus typhina* L. stem, including gallic acid, tryptophan, scopolin, methyl gallate, fustin, quercetin, rutin [63]. These constituents are largely responsible for the plant’s reported antioxidant, antimicrobial, anti-inflammatory, and cytotoxic activities. All these findings suggest that *R. typhina* may serve as a valuable source of natural bioactive compounds for different applications. *Rhus typhina* L. is a valuable plant that deserves further scientific study because of its many applications and therapeutic qualities. This species of sumac is also used in the food industry, in the making of fuels, cosmetics, and insecticides, as well as in the paper industry [64].

However, studies on their potential use as eco-friendly corrosion inhibitors have not yet been published; by electrochemical evaluation, only the weight loss measurement method was used to evaluate the corrosion inhibition efficacy of the *Rhus typhina* L. leaf extracts [65]. Răuță et al. [65] showed that the *Rhus typhina* L. leaf extracts, prepared in a 1:1 methanol–water mixture, exhibited excellent corrosion inhibition on mild steel, with efficiencies exceeding 99.9%. Extracts obtained from the summer leaves were more effective than those from the autumn leaves, as indicated by lower mass loss (0.0667 g vs. 0.0897 g), while the untreated control sample showed severe corrosion with a weight loss of 0.5823 g. Based on these findings, summer-collected leaves were selected for the present study.

According to [64], most of the active components are found in the leaves, which is one more reason why we selected this part of the plant for the present study.

This work focused on evaluating the environmental corrosion inhibitors from the *Rhus typhina* L. leaves extract (RTLE) on OL37 in 1 M HCl environments. High-performance liquid chromatography (HPLC) and liquid chromatography–mass spectrometry (LC-MS), were used to characterize the leaves extract. Open circuit potential (OCP), electrochemical impedance spectroscopy (EIS), and potentiodynamic polarization (PDP) were among the techniques used to assess the corrosion inhibition capacity of the RTLE. Surface morphological features were also examined using the scanning electron microscope (SEM), which is equipped with energy dispersive X-ray spectroscopy (EDX).

## 2. Results and Discussion

### 2.1. Quantitative and Qualitative Evaluation of Used Extract

**Figure 2:**
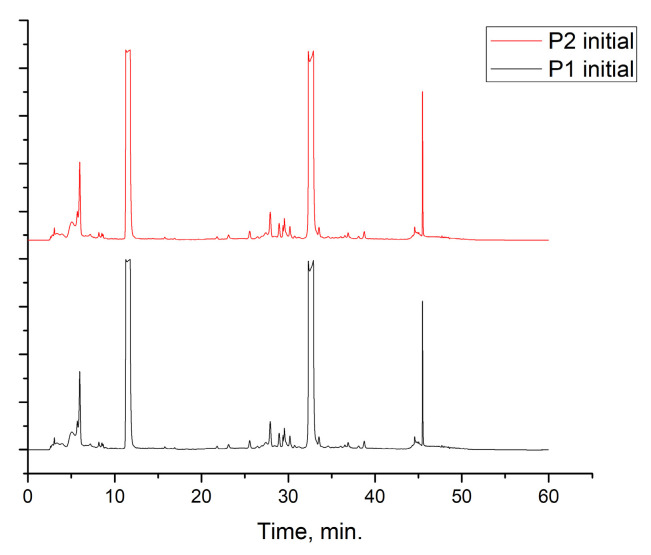
Chromatogram of the *Rhus typhina* L. leaf extracts.

The alcoholic and hydroalcoholic leaf extracts of the *Rhus typhina* L. composition were evaluated using the HPLC-DAD and LC-MS techniques (Table 2 and Figure 2). Beside a large number of compounds, like phenolic acids, there are three major compounds which mainly account for anticorrosion effects. Further, in this study, it will be noted as the P_1_ extract, for the methanolic extract of the *Rhus typhina* L. leaves, and as the P_2_ extract, for the hydroalcoholic (methanol and water (1:1 *v*/*v*) extract of the *Rhus typhina* L. leaves.

**Figure 3:**
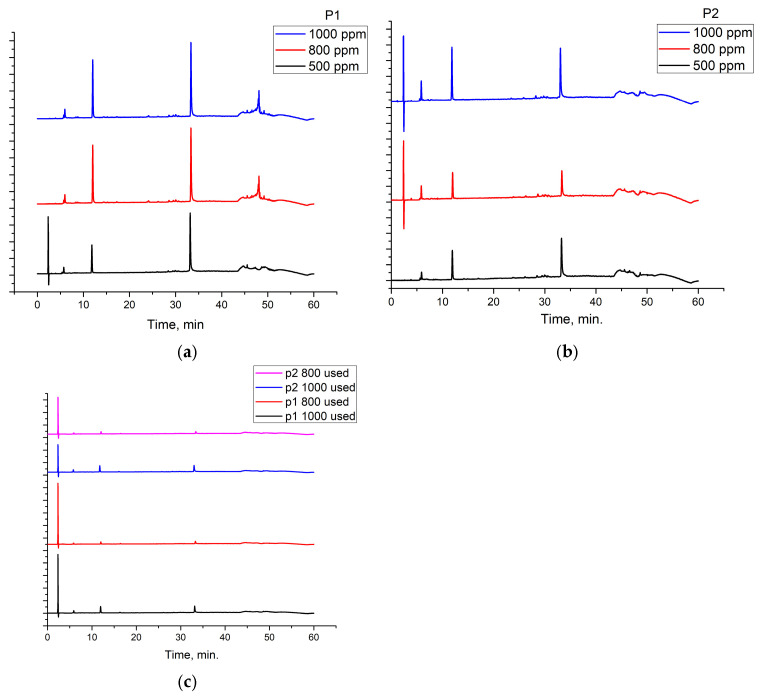
Chromatograms for the RTLE diluted in 1 M HCl solution used in the electrochemical process: (**a**,**b**) before and (**c**) after the corrosion inhibition.

The RTLE contain high quantities of phenolic acids and tannins. Not all compounds present in the extract can be identified and quantified even with the tandem HPLC-DAD/LC-MS. Only the major compounds were evaluated and quantified. Initial extracts are too concentrated to be used directly, thus, some dilutions were made in order to better asses the influence on natural compound concentrations. In Figure 3, the chromatograms of 1000, 800, and 500 ppm dilutions were presented before and after the electrochemical process. Initial extracts (Figure 2) were diluted as follow: for obtaining 1000 to 20 ppm solutions we take aliquots of each extracts (1000 to 20 mg) which were diluted to 1 L with 1 M HCl solution.

It can be observed that, in these chromatograms the working solution presents three major peaks which can be identified using the LC-MS technique (Table 3, Supplementary Materials).

Also, chromatograms for spent solutions (Figure 3c) show a significant reduction of the major compounds’ peak areas as a result of intense adsorption on the metal surface. The adsorption efficiency (Table 4) range is between 34 and 77% depending on the compound and the type of extract. The adsorption efficiency is significant taking into account that, in fact in this process, a competition between these compounds occur on the metal surface. For lower concentrations (presented in the Supplementary Materials), the adsorption efficiency reached 100% for all three compounds.

The adsorption efficiency was calculated as follows:

(1) $$Adsorption\;efficiency=\frac{{Peak\;area\;}_{bc}-{Peak\;area\;}_{ac}}{{Peak\;area\;}_{bc}}\times 100$$

where ${Peak\;area\;}_{bc}$—represents the peak area of each major compound found in the diluted extracts before corrosion process and ${Peak\;area\;}_{ac}$—represents the peak area of each major compound found in the diluted extracts after corrosion process. The peak area was used as a replacement for concentrations due to the absence of analytical standards for the two major compounds involved in the corrosion process.

Gallic acid and tannin compounds present an important anticorrosion effect due to the presence of numerous hydroxyl and carbonyl moieties. These groups contain oxygen atoms which are able to donate the lone pair of electrons in an interaction with the metal surface. Also, tannin molecules contain double bonds that are able to bind to electrons in the free orbitals of the metal atoms. Moreover, tannins feature large molecules which can cover the metal surface in a large proportion.

### 2.2. Electrochemical

#### 2.2.1. Potentiodynamic Polarization Technique

**Figure 4:**
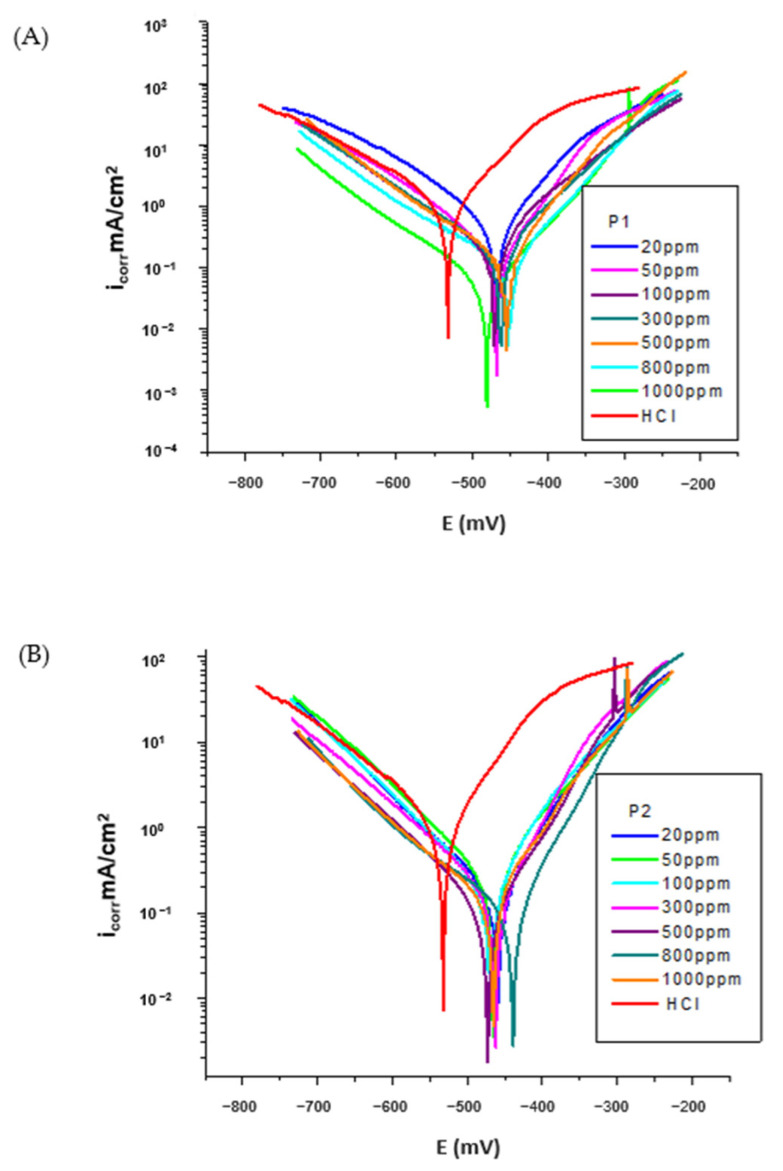
Polarization curve for OL37 in 1 M HCl at different concentrations of (**A**) P_1_ inhibitor at 25 °C and (**B**) P_2_ inhibitor at 25 °C.

Figure 4A,B shows the polarization curves of the OL37 electrode in 1 M HCl solution with and without different inhibitor concentrations, where the calomel electrode was used as a reference electrode for the analysis.

It was determined that both the cathodic and anodic polarization curves showed a lower current density in the presence of inhibitors than those determined in non-inhibitor solutions. This behavior showed that the corrosion inhibitors significantly suppressed both the cathodic and anodic reactions of the electrochemical processes, with a pronounced influence on the anodic reaction.

Also, in Figure 4A,B, it is observed that both the anodic process of metal dissolution and the cathodic process of hydrogen evolution were prevented by the addition of these environmentally friendly inhibitors in the aggressive medium (HCl).

#### 2.2.2. Corrosion Kinetic Parameters

As shown in the Tafel curves (Figure 4) and the corresponding data presented in Table 5 and Table 6, the corrosion kinetic parameters are categorized into measured and derived values. The experimentally measured parameters include the polarization resistance (R_p_), corrosion potential (E_corr_), the anodic and cathodic Tafel slopes (b_a_ and b_c_), and the corrosion current density (i_corr_). Based on these, the corrosion rates were subsequently calculated and expressed in terms of R_mpy_, penetration rate (P_mm/year_), and (K_g_). For each measurement the Tafel plot was carefully inspected, and the linear region closest to E_corr_ was selected and the linear curve was fitted (by the least squares method) using the VoltaMaster 4 software (v 7.09). The corrosion inhibition efficiency was calculated using Equation (1). The experimental results showed that the addition of these environmentally friendly inhibitors shifted the corrosion potential (E_corr_) to more positive values (increasing the corrosion potential (E_corr_) from −531 mV without inhibitor to −488 mV in the presence of the P_1_ extract and to −424 mV for the P_2_ extract, at 1000 ppm). The reduction of the inhibited anodic Tafel slopes (b_a_) shows the influence of the RTLE on the anodic dissolution process of the metal. This can be attributed to the adsorption of inhibitor molecules on the surface of OL37 and the prevention of corrosion by blocking the anodic dissolution process of the metal.

(2) $$E\;(\%)=\frac{{i_{corr}}_{0}-{i_{corr}}_{inh}}{{i_{corr}}_{0}}\times 100$$

where ${i_{corr}}_{0}$ is the corrosion current density at 0 ppm, without the RTLE and ${i_{corr}}_{inh}$ is the corrosion current density with the RTLE (20–1000 ppm).

The electrochemical data shows that the corrosion rate of the OL37 carbon steel in 1 M HCl solution significantly decreases with the addition of the green corrosion inhibitor (sample P_1_ RTLE). In the absence of the inhibitor (0 ppm), the corrosion current density (i_corr_) was high, at 0.855 µA·cm^−2^, and the polarization resistance (R_p_) was very low (20 Ω·cm^−2^), indicating severe corrosion. As the concentration of the inhibitor increased from 20 ppm to 1000 ppm, i_corr_ progressively decreased to 0.059 mA·cm^−2^, at 1000 ppm P_1_, while R_p_ rose suddenly to 243 Ω·cm^−2^, confirming improved surface protection.

At all concentrations, the corrosion rate, determined by different measures (P_mm_/year, g/m²·h), dramatically decreased. The shift in E_corr_ is less than 85 mV, indicating that the compound acts as a mixed-type inhibitor with a more pronounced effect on the anodic reaction. Changes in the Tafel slopes (b_a_ and b_c_) further support that both anodic dissolution and cathodic hydrogen evolution were suppressed, confirming the inhibitor’s dual-action mechanism.

In the absence of the inhibitor, the corrosion current density was highest at 0.855 mA/cm^2^, indicating active corrosion. The low polarization resistance (20 Ω·cm^−2^) confirms the high corrosion rate (399 mpy). This establishes the aggressive corrosive nature of 1 M HCl toward the OL37 steel, and serves as the baseline for comparison. The addition of the P_2_ RTLE inhibitor significantly reduces i_corr_ and R_mpy_, and increases R_p_, demonstrating its corrosion-inhibiting action. For instance, at 20 ppm, i_corr_ dropped to 0.183 mA/cm^2^, representing a 79% inhibition efficiency. This trend continues up to 1000 ppm, where i_corr_ reached a minimum of 0.055 mA/cm^2^, and R_p_ peaked at 236 Ω·cm^−2^, corresponding to the maximum inhibition efficiency of 94%.

With increasing concentrations of the P_2_ extract, the corrosion potential showed slight shifts towards more positive values (from −531 mV at 0 ppm to −464 mV at 20 ppm and −440 mV at 800 ppm). However, these shifts are less than 85 mV, indicating that the P_2_ extract acts as a mixed-type inhibitor—affecting both anodic and cathodic reactions.

The anodic (b_a_) and cathodic (b_c_) Tafel slopes show variations but no systematic trend, suggesting that the inhibition mechanism is not purely controlled by changes in the activation energy but rather by surface coverage and adsorption of the inhibitor molecules on both the anodic and cathodic sites.

By comparative investigation of the inhibition efficiency and corrosion rate (expressed in R_mpy_, P, and K_g_) for all inhibitors, under the same conditions, it is evident that these potential P_1_ and P_2_ inhibitors have excellent efficiency for corrosion protection of the OL37 electrode in 1 M HCl.

#### 2.2.3. Immersion Time

**Figure 5:**
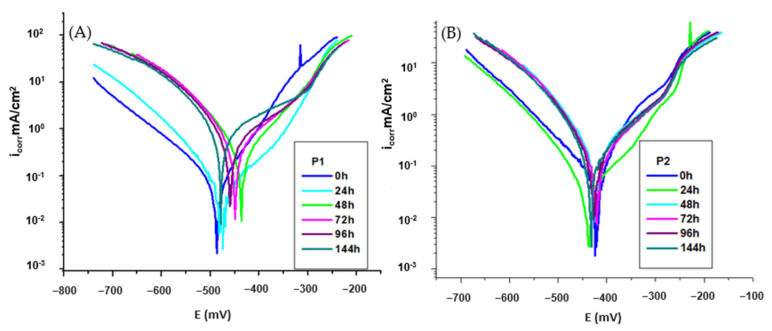
Polarization curve for OL37 in 1 M HCl at different immersion time at 25 °C for (**A**) P_1_ inhibitor and (**B**) P_2_ inhibitor.

The influence of increasing the immersion period 0–144 h on the corrosion inhibition of the organic P_1_ and P_2_ compounds on the corrosion of OL37 in 1 M HCl was analyzed by potentiodynamic polarization. The influence of the protective efficacy of these inhibitors by immersion periods is presented in Figure 5A,B and Table 7 and Table 8.

Despite this, after 96 h of immersion, the inhibition efficiency of the P_2_ extract remains at 88% (Figure 5B and Table 7) demonstrating that the P_2_ extract is a highly effective inhibitor over extended exposure periods. It presented a non-degradable behavior after 96 h of immersion in the aggressive medium, further verifying its excellent protective capacity. By contrast, as shown in Figure 5A and Table 6, the inhibition efficiency of the P_1_ extract drops to 53% after the same immersion period, indicating that the P_1_ extract is significantly less effective as a long-term corrosion inhibitor.

#### 2.2.4. Electrochemical Impedance Spectroscopy

**Figure 6:**
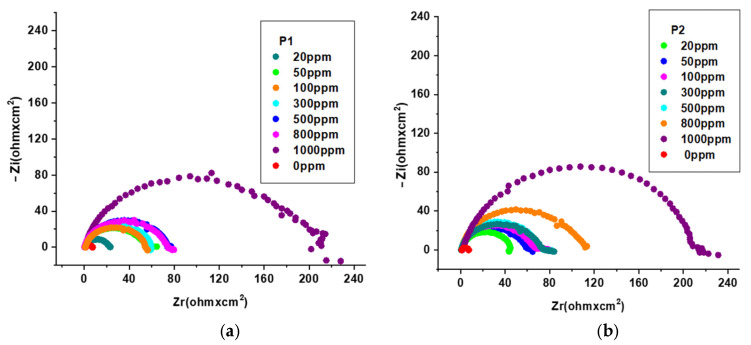
Nyquist plot for OL37 in 1 M HCl at different inhibitor concentrations at 25 °C for (**a**) P_1_ and (**b**) P_2_.

The corrosion-inhibiting effect of the OL37 steel with environmentally friendly inhibitors was also studied by electrochemical impedance spectroscopy (EIS). The EIS tests present information about the protective properties of these green inhibitors on the OL37 electrodes in the corrosive-HCl environment. Nyquist plots for OL37 uninhibited and in the presence of different inhibitor concentrations are shown in Figure 6.

Figure 6a,b shows that the diameters of the capacitance loops in the presence of the P_1_ and P_2_ extracts are larger than those in the absence, assuming that these inhibitors have excellent protective properties on the OL37 electrode in the 1 M HCl medium.

It is evident from the Nyquist plots that the impedance response of the carbon steel was significantly modified by the addition of the P_1_ and P_2_ inhibitors, implying that the protective layer obtained was confirmed by the addition of these two RTLEs.

**Figure 7:**
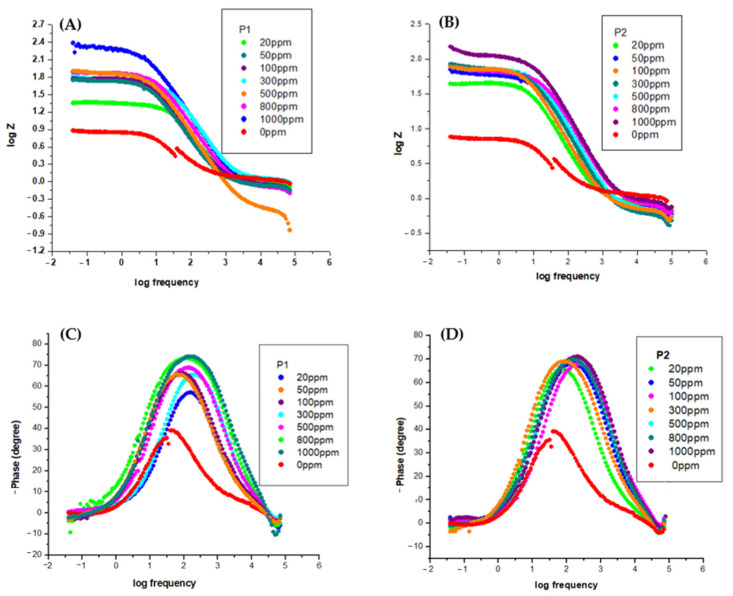
Bode plot for OL37 in 1 M HCl at different inhibitor concentrations at 25 °C for P_1_ (**A**,**C**) and P_2_ (**B**,**D**).

The Bode plots shown in Figure 7 are consistent with the Nyquist plots (Figure 6). It is evident that, without the inhibitor, the OL37 electrode denotes a time constant suitable for a phase angle of 35°; that is, it exhibits capacitive behavior.

From Figure 7, it can be seen that the presence of the P_1_ and P_2_ RTLE on the “phase angle versus logarithm of frequency” plot shows a very well established maximum attributed to a phase angle of about 75°. Therefore, in this case, the electrode has a higher capacitive behavior in agreement with the Nyquist plots and the experimental data obtained by potentiodynamic polarization.

**Figure 8:**
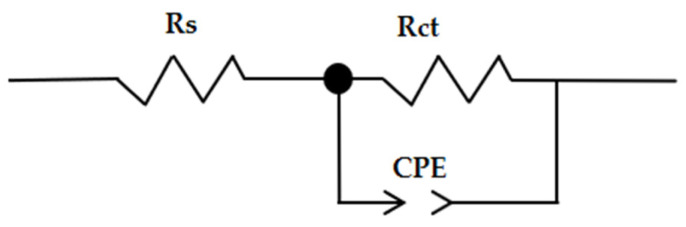
The equivalent circuit.

The analysis of the experimental data was determined by matching the results (splicing the experimental results) to obtain a suitable equivalent circuit shown in Figure 8.

The various impedance characteristics, such as solution resistance (Rs), charge transfer resistance (Rct), and double layer capacitance (Cdl), were calculated at the temperature of 25 °C; the results are shown in Table 9 and Table 10.

Electrochemical impedance spectroscopy (EIS) was employed to further evaluate the corrosion inhibition performance of the P_1_ and P_2_ inhibitors on the OL37 carbon steel in 1 M HCl at 25 °C. The Nyquist plots were fitted using an equivalent circuit model; the extracted parameters are summarized in Table 9 and Table 10. In the absence of inhibitors, the steel exhibited a low charge transfer resistance (Rct) of 6.32 Ω·cm^2^, indicative of high corrosion activity. Upon the addition of inhibitors, a substantial increase in Rct was observed, reflecting enhanced corrosion protection. Specifically, the Rct increased to 214 Ω·cm^2^ for the P_1_ extract and 223 Ω·cm^2^ for the P_2_ extract at a concentration of 1000 ppm, confirming the superior performance of the P_2_ extract in forming a more resistive protective layer.

Simultaneously, the double-layer capacitance, represented by the constant phase element (Q-Yo), decreased significantly with the increasing inhibitor concentration, suggesting the adsorption of the inhibitor molecules on the metal surface and a consequent reduction in the active surface area available for the charge transfer. The phase shift factor (Q-n) increased from 0.74 in the uninhibited solution to values between 0.88 and 0.90 in the presence of the inhibitors, indicating a transition towards a more homogeneous and capacitive surface.

The EIS results show that the charge transfers resistance (Rct) increased and the double layer capacitance (Cdl) decreased with the concentration of the inhibitor. As a consequence of the increase of the Rct values with the inhibitor concentration, the inhibition effectiveness increases significantly, which demonstrates that the inhibitor exhibits good protective ability on the OL37 electrode corrosion.

Therefore, under these circumstances, the inhibited electrodes have good capacitive behavior according to the Nyquist data and the determinations carried out by the potentiodynamic polarization technique. The increase in the impedance modulus (Zmod) indicates an important protective capacitance, and it is evident that the Zmod increases when the inhibitor film is enhanced. Higher Zmod leads to higher protective efficacy.

**Figure 9:**
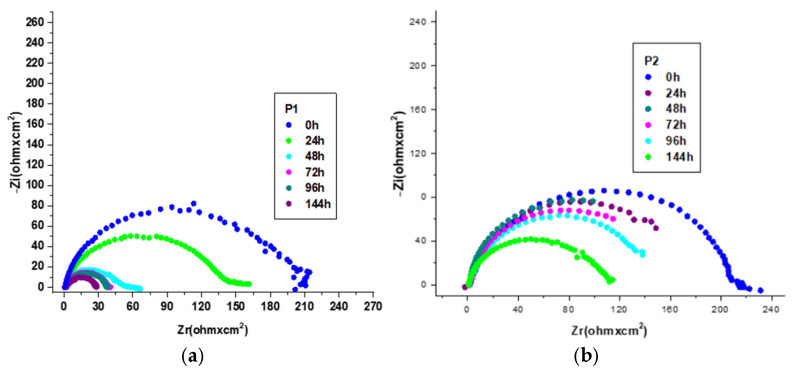
Nyquist plot for OL37 in 1 M HCl at 1000 ppm inhibitor concentrations at different immersion times at 25 °C for (**a**) P_1_ and (**b**) P_2_.

The effect of increasing the immersion period (0–144 h) on the corrosion inhibition performance of the environmentally friendly P_1_ and P_2_ inhibitors (at 1000 ppm) on the OL37 steel in 1 M HCl was evaluated using the EIS. The results illustrating the protective properties of these inhibitors over time are presented in Figure 9 and Table 11 and Table 12.

As shown in Figure 9, the size of the capacitance loops in the presence of the P_1_ and P_2_ inhibitors decreases slightly with increasing immersion time. Moreover, between 0 and 144 h of immersion, a slight reduction in the protective efficiency of the inhibitor film is observed, likely due to the diffusion of corrosive chloride ions (Cl^−^) through the inhibited surface. The EIS results (Figure 9 and Table 11 and Table 12) confirm the ability of the environmentally friendly P_1_ and P_2_ inhibitors to impede the migration of aggressive chloride ions (Cl^−^) to the metal surface over extended immersion periods. After 96 h of immersion, the inhibition performance of the P_2_ extract remains stable, demonstrating its effectiveness and durability as a long-term corrosion inhibitor. Its non-degradable behavior in the corrosive medium further confirms its good protective capacity. By contrast, as shown in Figure 9 and Table 11, the inhibition performance of the P_1_ extract begins to decline after 72 h of immersion, indicating that the P_1_ extract is significantly less effective for long-term corrosion protection.

#### 2.2.5. Adsorption Isotherm

**Figure 10:**
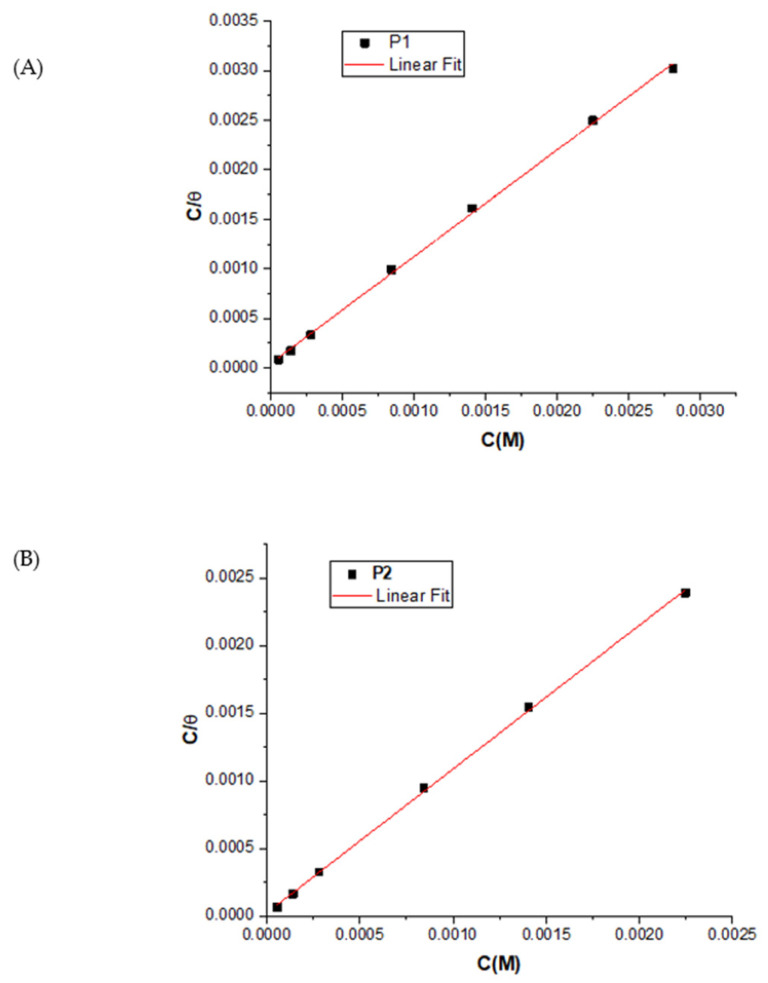
Langmuir plot for P_1_ (**A**) and (**B**) P_2_ on OL37 in 1 M HCl.

The adsorption isotherm elucidates the interaction between the organic inhibitor and the OL37 surface, with its protective efficiency attributed to adsorption. The inhibitor’s adsorption is considered the primary corrosion prevention mechanism [66]. To assess the influence of the inhibitor concentration, the data were fitted to the Langmuir isotherm (Figure 10), which showed an excellent correlation with surface coverage, confirming monolayer adsorption behavior.

The Langmuir adsorption isotherm is used to describe the inhibitor’s adsorption mechanism, and is expressed as follows:

(3) θ/(1−θ)=K×C

where C is the inhibitor concentration, θ is the surface coverage, and K is the adsorption equilibrium constant. Surface coverage (θ) is calculated from electrochemical measurements using the following relation:

θ = (i_corr_ − i_inh_)/i_corr_ (4)

where i_corr_ and i_inh_ represent the corrosion current densities in 1 M HCl without and with the inhibitor, respectively.

All correlation coefficients (R^2^ > 0.99), such as P_1_ (R^2^ = 0.9986) and P_2_ (R^2^ = 0.9996), confirm that the corrosion inhibition is primarily due to the adsorption of the inhibitors onto the substrate. The initial step in the corrosion inhibition process of the metal substrate in 1 M HCl involves the interaction between the inhibitor molecules and the metal surface, as follows:

(5) $$Me+INH↔{Me\left(INH\right)}_{ads}\;↔\;{Me}^{n+}+ne^{-}+INH\;(\mathrm{Me}=\mathrm{Fe},\;\mathrm{INH}=P_{1},\;P_{2})$$

In this study, linear plots of C_inh_/θ versus C_inh_ with a slope close to 1 (slope for the P_1_ extract is 1.07, and for the P_2_ extract is 1.05) confirm that the adsorption of the green inhibitors follows the Langmuir isotherm. The adsorption equilibrium constant (K_ads_), calculated from the intercept (Table 13), reflects the strength of the adsorption. High K_ads_ values indicate strong surface interaction, effective corrosion inhibition by the P_1_ and P_2_ extracts in 1 M HCl, and significant modification of the metal–solution interface, enhancing the corrosion resistance of the OL37 steel.

The adsorption equilibrium constant (K_ads_) is related to the standard free energy of adsorption ($∆G_{ads}^{{}^{\circ}}$) through the following relationship:

(6) ln⁡Kads = −∆Gads°RT

(7) $$∆G_{ads}^{{}^{\circ}}=-RT\ln\left(55.5K_{ads}\right)$$

where 55.5 mol/L is the molar concentration of water substituted with 1000 g/L to meet the unit of K_ads_, R is the universal gas constant, and T is the thermodynamic temperature of the medium.

The negative $∆G_{ads}^{{}^{\circ}}$ values indicate that the adsorption of the green inhibitors is spontaneous, with stronger interactions between the inhibitor molecules and the substrate. The $∆G_{ads}^{{}^{\circ}}$ values around −20 kJ/mol suggest physical adsorption (electrostatic interaction), while values near −40 kJ/mol or lower indicate chemisorption, where charge sharing or coordination bonds form between the OL37 substrate and the inhibitor molecules (Table 13).

#### 2.2.6. The Reaction Mechanism

**Figure 11:**
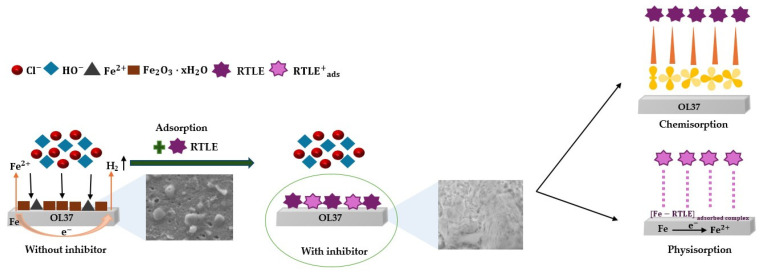
Schematic representation of the metal surface with adsorption of the main phytochemical constituents from the RTLE as the GCI on the OL37 carbon steel surface, forming a protective film; without the RTLE as the GCI, the surface corrodes.

Presented below is a detailed overview of the chemical reactions involved in the corrosion process of carbon steel (which contains 99.293% Fe) in acidic environments (in HCl), and how this process can be inhibited by green inhibitors via chemisorption mechanisms, with a schematic representation in Figure 11. The reaction mechanism has been adapted from [34,67,68], and is written below:

General Corrosion Mechanism:

Anodic process: metal dissolution, iron oxidation

(a) Without Inhibitor (Acidic medium):
  (8) $$Fe+H_{2}O↔{FeOH}_{ads}+H^{+}+e^{-}$$
  (9) $${FeOH}_{ads}\;↔\;{{FeOH}^{+}}_{ads}+e^{-}\;(\mathrm{rate}\text{-}\mathrm{determining}\;\mathrm{step})$$
  (10) $${FeOH}^{+}+H^{+}\;↔\;{Fe}^{2+}+H_{2}O$$
(b) In the presence of ${Cl}^{-}$ ions:
  (11) $$Fe\;+H_{2}O+\;{Cl}^{-}↔\;{FeClOH}_{ads}+H^{+\;}+\;e^{-}$$
  (12) $${FeClOH}_{ads}\;↔\;{{FeClOH}^{+}}_{ads}+e^{-}\;(\mathrm{rate}\text{-}\mathrm{determining}\;\mathrm{step})$$
  (13) $${{FeClOH}^{+}}_{ads}+H^{+\;}\;↔\;{Fe}^{2+\;}+{Cl}^{-}+H_{2}O$$
  (14) $${Fe}^{2+\;}+\;{2Cl}^{-}\;\to \;{FeCl}_{2}$$
  (15) $${Fe}^{2+\;}\to \;{Fe}^{3+\;}+e^{-}\;$$

The acidic medium accelerates the anodic reaction, increasing the corrosion rate.

Cathodic process: reduction of oxygen, hydrogen evolution mechanism

(16) $$2H^{+}\left(aq\right)+{2e}^{-}\to H_{2}(g)$$

(17) $${Fe}^{2}+O_{2}+{6H}_{2}O\to {4Fe}^{3+}+4{OH}^{-}$$

(18) $${4Fe}^{2+}+{3H}_{2}O\to Fe(O{H)}_{3}↓(\mathrm{insoluble})$$

Over time, $Fe(O{H)}_{3}$ dehydrates and transforms into the following:

(19) $$4Fe+3O_{2}+{3H}_{2}O\to 4Fe(O{H)}_{3}\to {Fe}_{2}O_{3}\cdot xH_{2}O\;(\mathrm{rust})$$

(20) $$\mathrm{Chemical}\;\mathrm{reactions}:\;{Fe}^{2+}+2{HO}^{-}\;\to Fe(O{H)}_{2}\to \;Fe(O{H)}_{3}\to {Fe}_{2}O_{3}$$

Corrosion Inhibition Mechanism (with RTLE—inhibitor):

(21) $$Fe\;+H_{2}O\;↔{{FeH}_{2}O}_{ads}$$

(22) $${{FeH}_{2}O}_{ads}+RTLE\;↔\;{{FeOH}^{-}}_{ads}+\;H^{+\;}+RTLE$$

(23) $${{FeH}_{2}O}_{ads}+RTLE\;\to \;{{FeOH}^{-}}_{ads}+\;H_{2}O$$

(24) $${{FeOH}^{-}}_{ads}\to \;{FeOH}_{ads}+\;e^{-}\;(\mathrm{rate}\text{-}\mathrm{determining}\;\mathrm{step})$$

(25) $${Fe-RTLE}_{ads}\;\to \;{{Fe-RTLE}^{+}}_{ads\;}+\;e^{-}$$

(26) $${FeOH}_{ads}+\;{{Fe-RTLE}^{+}}_{ads\;}↔\;{Fe-RTLE}_{ads}+{FeHO}^{+}$$

(27) $${FeHO}^{+}+H^{+\;}↔\;{Fe}^{2+\;}+H_{2}O$$

(28) $$RTLE\;+\;{Fe}^{2+\;}\to \;{[Fe-RTLE]\;}_{adsorbed\;complex}$$

(29) $${Fe}^{2+\;}+\;R-OH\;\to Fe-O-R\;(chelate\;complex)\;R\text{-}\mathrm{OH}\;(\mathrm{phenolic}\;\mathrm{group})$$

### 2.3. SEM and EDX Analysis

**Figure 12:**
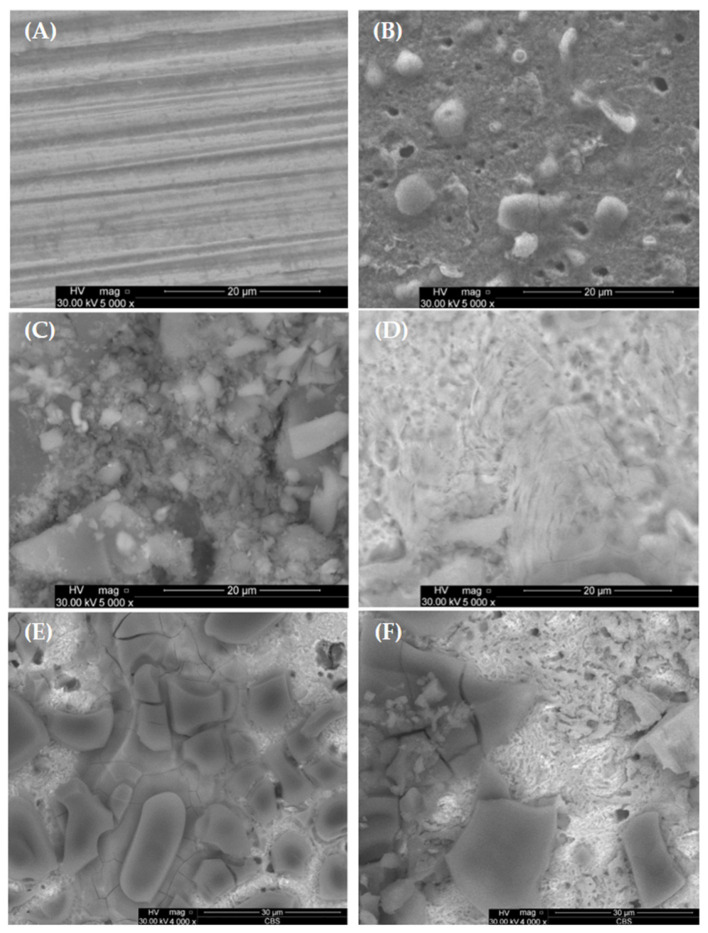
SEM morphology of carbon steel surface: (**A**) freshly polished carbon steel; (**B**) corrosion products in HCl without RTLE; (**C**) with RTLE, P_1_ 1000 ppm; (**D**) with RTLE P_2_ 1000 ppm; (**E**) RTLE, P_1_ 1000 ppm after 96 h immersion time; (**F**) RTLE P_2_, 1000 ppm after 96 h immersion time.

Figure 12 displays a scanning electron microscopy (SEM) image illustrating the freshly polished carbon steel (Figure 12A), carbon steel with corrosion products in HCl without inhibitor (Figure 12B), with corrosion inhibitor P_1_ (Figure 12C) and P_2_ (Figure 12D). The surface of the corroded carbon steel is irregular and has more imperfections than the carbon steel, which is inhibited, as can be seen visually. *Rhus typhina* L. successfully protects the carbon steel in acidic solutions including HCl.

**Figure 13:**
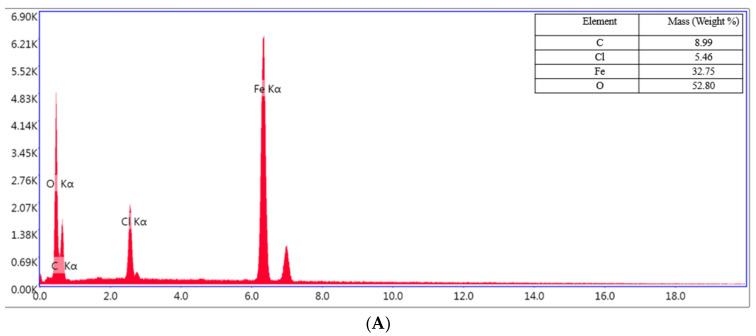

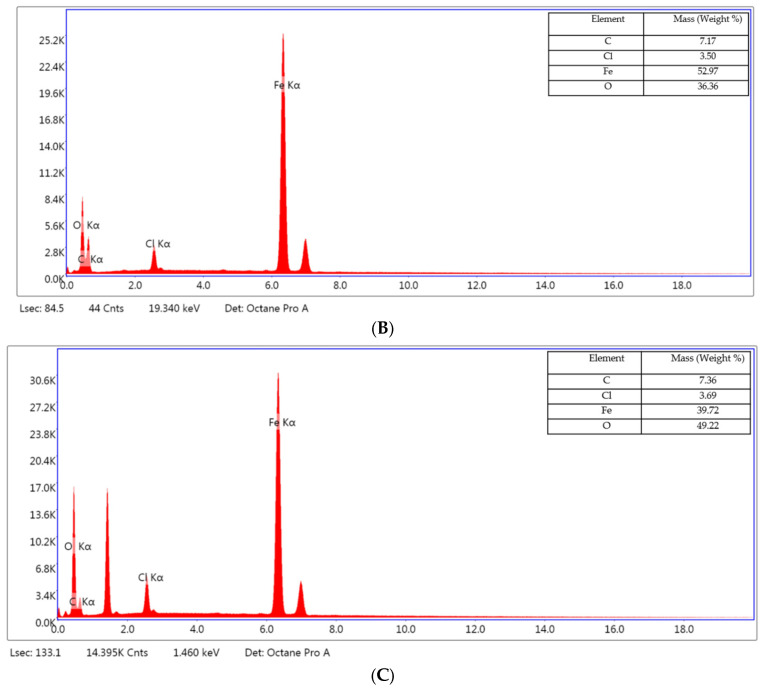
EDS spectrum for the OL37 uninhibited electrode (**A**), and protected electrodes with 1000 ppm of P_1_ (**B**) and P_2_ (**C**).

A protective layer that forms on the carbon steel plate’s surface can significantly slow down the rate of corrosion, creating a protected metal surface with less surface roughness. The complete surface shown in Figure 12C,D was subjected to the EDX analysis, and Figure 12 and Figure 13 compiles and summarizes the results.

After 96 h of immersion, the SEM analysis (Figure 12E,F—where E is the OL37 electrode surface with the RTLE, P_1_, 1000 ppm after 96 h immersion time, and F is the OL37 electrode surface with the RTLE, P_2_, 1000 ppm after 96 h immersion time) shows that the surface treated with the P_2_ inhibitor exhibits significantly less surface degradation compared to the surface protected with the P_1_ inhibitor. This finding, in agreement with the electrochemical results, confirms that the P_2_ inhibitor maintains a more effective and stable corrosion inhibition over time in acidic conditions than the P_1_ inhibitor.

The EDX spectrum of the OL37 carbon steel sample immersed in the uninhibited solution reveals characteristic peaks corresponding to a significant presence of chloride (Cl) and oxygen (O) elements (Figure 13A). These elements are associated with the formation of corrosion products on the steel surface. However, upon the addition of the eco-friendly P_1_ and P_2_ inhibitors, the concentrations of these elements are reduced, indicating the inhibitors’ protective efficacy against corrosive attack (Figure 13B,C). The inhibitors function by limiting chloride ion adsorption and preventing iron oxidation at the metal surface. In the present study, it is evident that, in the presence of hydrochloric acid, elevated levels of chlorine and oxygen accelerate the corrosion process. Conversely, in the presence of the P_1_ and P_2_ inhibitors (extracted from the RTLE), a marked reduction in these elements is observed. This effect is attributed to the formation of a protective film on the steel surface which mitigates corrosion.

## 3. Materials and Methods

### 3.1. Extract Preparation and Leaves Extraction

**Figure 14:**
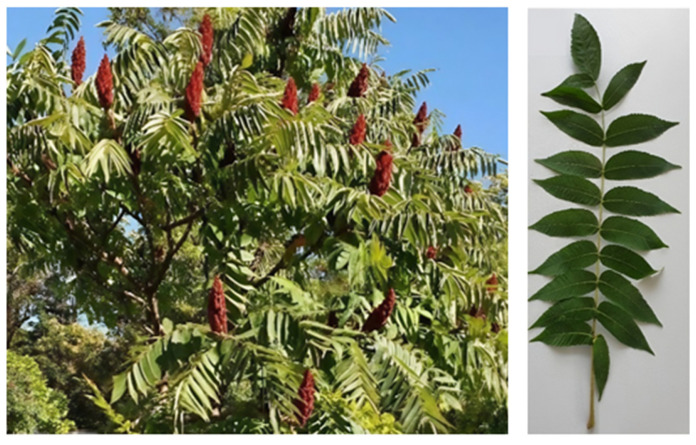
*Rhus typhina* L. plant (**left**) and the plant material for the extract (**right**).

**Figure 15:**
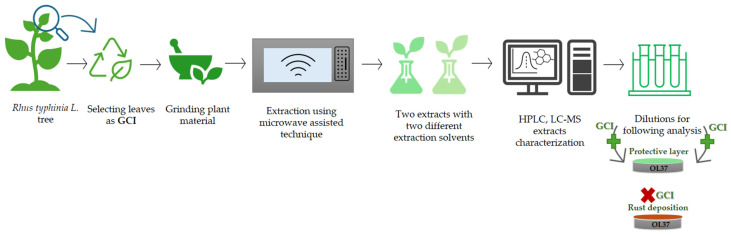
Schematic representation of the extraction process, characterization and application of the RTLE as green corrosion inhibitors for OL37.

The leaves were collected from the local domestic areas in the summer season (Figure 14). The leaves were non-chemically cleaned, left at room temperature under controlled environmental conditions. The dried vegetal material was evenly shred before the extraction processes began in order to obtain pieces smaller than 2 mm (as verified through screening). Schematic representation of the extraction process, characterization and application of the RTLE as green corrosion inhibitors is illustrated in Figure 15.

The method used to obtain the natural extracts was a microwave-assisted method that involved heating the vegetal material and the solvent for 60 min at 100 °C with an Ethos Easy Advanced Microwave Digestion System (Milestone S.r.l,, Sorisole, Italy) using an 800 W microwave power. The solvents for the extraction were methanol and ultra-pure water.

The ratio of vegetable material to solvent was kept at 1:10 (*w*/*v*), and the solvent included the following:

- Methanol, 100%, noted as P_1_;
- Hydroalcoholic mixture of methanol and water (1:1 *v*/*v*), noted as P_2_.

Reagent-quality methanol was utilized (Sigma Aldrich, St. Louis, MO, USA), and laboratory-sourced bi-distilled water (GFL 2102 water still, GFL, Burgwedel, Germany) was used for all tests.

### 3.2. High-Performance Liquid Chromatography (HPLC) Extracts Characterization

For the HPLC mobile phase, we used trifluoroacetic acid, 0.1% (Sigma Aldrich), acetonitrile (Sigma Aldrich), and distilled water. An L-3000 HPLC system (Rigol Technologies Inc., Beijing, China) with a diode-array detector (HPLC-DAD) and a Kinetex EVO C18, 150 × 4.6 mm, 5 µm particle size, Kinetex EVO C18 column (Phenomenex, Torrance, CA, USA) was used to quantify the compounds contained in the extracts. A two-solvent system made up the mobile phase, and a gradient mode elution was used. An amount of 0.1% trifluoroacetic acid (TFA) in water (I) and 0.1% TFA in acetonitrile (II) were the solvents utilized. The following was the elution gradient: 2–100% solvent (II) for 60 min at 35 °C with a 0.6 mL/min elution flow rate. In accordance with the literature, the analysis was carried out at five distinct wavelengths (255, 280, 325, and 355 nm). The injection volume was 10 μL. The LC-MS conditions were as follows: Column Thermo Accord C18 (150 × 4.1 mm, 2.1 μm); Pump operating conditions: gradient; Mobile phase A: water with 0.1% formic acid; Mobile phase B: methanol with 0.1% formic acid and 4 mM ammonium formate; Flow rate: 0.5 mL/min; column temperature: 30 °C; injection volume: 5 μL. Mass spectrometry conditions were as follows: Ion Source: Heated electrospray (HESI-II); Ion Mode: Positive; Capillary Temperature: 280 °C; Vaporizer Temperature: 295 °C; Spray Voltage: 2200 V; Sheath Gas: 32 arbitrary units; Aux Gas: 7 arbitrary units; Scan Type: Full MS scan; Mass Range: *m*/*z* 120–1000; Mass Resolution: 70,000

Stock solutions with the reference chemicals from several classes: The following substances were prepared to have a concentration of 1000 µg/mL: phenolic acids (gallic acid); flavonoids (catechin, epicatechin, hyperoside, naringin, naringenin); hydroxycinnamic acids and derivatives (caffeic acid, p-coumaric acid); chlorogenic acids (chlorogenic acid). The concentrations utilized for calibration curves ranged from 10 to 400 µg/mL. At each retention time, the identification and quantification of the compound was accomplished by a comparison with the standard spectra.

### 3.3. Electrochemical Studies

The working electrode for the corrosion investigation was OL37. The structure of OL37 is represented in Table 14.

**Figure 16:**
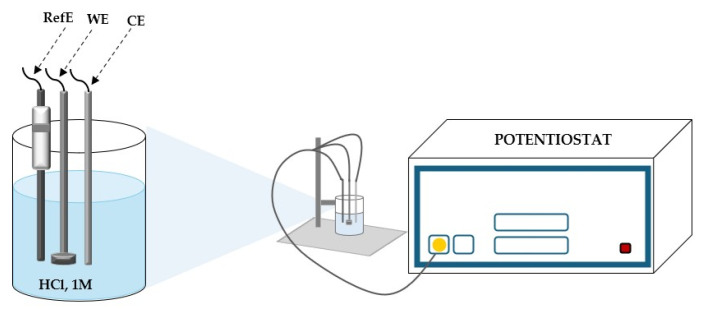
Diagram of the electrochemical cell, in 1 M HCl (RefE = reference electrode; WE = working electrode OL37; CE = platinum sheet counter electrode).

A standard three-electrode cell setup was used for the tests, with a disk electrode serving as the operational working electrode (Figure 16). A calomel electrode acted as the reference, while a platinum plate served as the opposing electrode next to it. To obtain a mirror-like quality, the working samples (carbon steel) were mechanically polished using sandpapers with different grits (600–4000). After being washed with benzene to remove any oily residue, the electrodes were rinsed with bi-distilled water, allowed to dry at room temperature, and then put into the electrochemical cell.

The corrosive medium was 1 M HCl, achieved by diluting AG 36% HCl (from Merck, Rahway, NJ, USA) with bi-distilled water.

The corrosion inhibition efficiency of the P_1_ and P_2_ compounds was investigated for the OL37 carbon steel immersed in the 1 M HCl solution. Electrochemical measurements were carried out using a PGZ 301 potentiostat/galvanostat controlled by VoltaMaster 4 software.

The efficacy of these inhibitors was investigated by potentiostatic and potentiodynamic polarization and electrochemical impedance spectroscopy (EIS). The evaluation of the inhibitor performance was primarily based on the polarization studies and EIS analysis. Experimental impedance determinations were performed at open-circuit potential-OCP in the frequency range from 100 KHz to 40 mHz with an AC wave of ± 10 mV (peak-to-peak).

The results underscore the effectiveness of the corrosion inhibitors as a reliable method for mitigating metal degradation in aggressive media, by suppressing anodic, cathodic, or both electrochemical reactions involved in the corrosion process.

Examination of the Tafel polarization curves was carried out by plotting the potential from cathodic to anodic potential in the potential range of ±250 mV versus OCP, at a sweep rate of 2 mVs^−1^ for the working OL37 electrode.

For the electrochemical analysis, dilutions were made from the stock extract solution (solvent + natural compounds, P_1_ and P_2_), resulting in the following concentrations for each sample: 20, 50, 100, 300, 500, 800, and 1000 ppm.

### 3.4. Surface Analysis

An extremely effective method for examining the modifications brought on by corrosion on the electrode surface is surface analysis. Consequently, energy dispersive X-ray spectroscopy (EDX) and scanning electron microscopy were used to examine the surface of the OL37 samples. The electron microscopy images were obtained using a Quanta Inspect F50 (FEI Company, Eindhoven, The Netherlands) equipped with a field emission gun (FEG) with a 1.2 nm resolution, and an energy dispersive X-ray spectrometer (EDX) with an MnK resolution of 133 eV Kα.

## 4. Conclusions

This study demonstrates that the methanolic (P_1_) and hydroalcoholic (P_2_) extracts of the *Rhus typhina* L. leaves are effective green inhibitors for the corrosion of the OL37 carbon steel in 1 M HCl solution. Electrochemical analyses, potentiodynamic polarization, and electrochemical impedance spectroscopy showed that both extracts significantly reduced corrosion rates, with efficiencies reaching 93% for the P_1_ extract and 94% for the P_2_ extract at 1000 ppm. These results correlate with increases in polarization resistance and reductions in corrosion current density, confirming a concentration-dependent protective effect.

The observed shift in corrosion potential and surface coverage values suggests that both extracts act as mixed-type inhibitors, with a stronger influence on anodic processes. HPLC profiling identified a variety of phytochemicals, including gallic, caffeic, ferulic, and sinapic acids, compounds known for their antioxidant and metal-chelating properties. These constituents likely adsorb onto the steel surface via a combined physisorption–chemisorption mechanism, forming a barrier that inhibits electron transfer and ion penetration.

Surface analyses (SEM/EDX) confirmed the formation of a continuous, compact protective layer, with the P_2_ sample showing superior long-term stability and lower morphological degradation after 96 h of immersion. These findings were further supported by Langmuir isotherm modeling, which indicates monolayer adsorption and suggests strong interactions between the inhibitors and the metal substrate.

Overall, the *Rhus typhina* L. leaf extracts, particularly the P_2_ extract, demonstrate high potential as sustainable, environmentally friendly alternatives to conventional corrosion inhibitors. Future research should explore their performance under extended immersion times, elevated temperatures, and in other corrosive media, such as sulfuric acid or saline environments. Additionally, expanding the application to other metal substrates and integrating advanced techniques, such as XPS and AFM, would provide deeper insight into the adsorption mechanisms involved.

## Figures and Tables

**Table 1:** The latest examples of plant part extracts acting as green corrosion inhibitors on various metal surfaces and in diverse corrosive environments.

Nr.crt.	Extract	Extract Concentration	Conditions	Metal Surface	Efficiency	Ref.
1.	*Andrographis paniculata* leaf	400 ppm	H_2_SO_4_	Mild steel	95.14%	[46]
2.	*Michelia alba* leaf	not mentioned	H_2_SO_4_	Cu	94.72%	[47]
3.	*Araucaria heterophylla* leaf	1000 ppm	HCl	Mild steel	83.94%	[48]
4.	*Syzygium polyanthum* (Wight) Walp. leaf	2000 ppm	HCl	Carbon steel	94.65 ± 0.94%	[49]
5.	*Natal Plum* leaf	2.5 g/L	HNO_3_	Brass alloy	98%	[50]
6.	*Boehmeria nivea* (L.) Gaudich leaf	1000 mg L^−1^	HCl	Mild steel	93.4%	[51]
7.	*Pistia stratiotes* leaf	400 ppm	HCl	Mild steel	93.7%	[52]
8.	*Atriplex halimus* L. leaf	600 mg/L	HCl	Carbon steel	95.1%	[53]
9.	*Dillenia suffruticosa* leaves	1000 ppm	HCl	Mild steel	81.4%	[54]

**Table 2:** Concentration of some compounds found in the RTLE.

Compound	P_1_ Initial (mg/L)	P_2_ Initial (mg/L)
Gallic acid	96.86	71.09
Protocatehuic acid	2.79	2.09
Caffeic acid	35.68	27.30
Chlorogenic acid	5.96	16.64
Syringic acid	13.32	9.32
Ferulic acid	130.57	193.99
Sinapic acid	381.69	397.98
Ellagic acid	190.44	179.20
Rosmarinic acid	35.96	29.22

**Table 3:** Major compounds found in the RTLE involved in the anticorrosion process.

Retention Time (min.)	Compound Name	Chemical Structure
5.90	Gallic acid	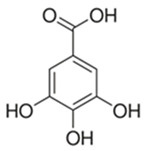
11.87	3-O-methylgallic acid	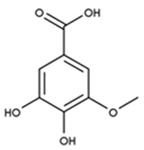
33.14	beta-1,2,3,4,6-penta-O-galloyl-D-glucopyranose	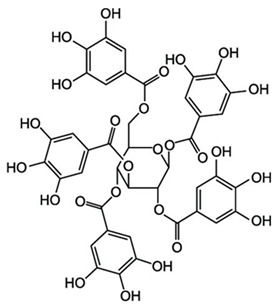

**Table 4:** Adsorption efficiency of natural compounds present in extract on metal surface.

Sample	Adsorption Efficiency (%)
Gallic acid	
P_1_ 1000	58.84
P_2_ 1000	73.73
P_1_ 800	34.09
P_2_ 800	52.97
3-O-methylgallic acid	
P_1_ 1000	59.66
P_2_ 1000	72.77
P_1_ 800	31.49
P_2_ 800	50.30
beta-1,2,3,4,6-penta-O-galloyl-D-glucopyranose	
P_1_ 1000	67.45
P_2_ 1000	77.65
P_1_ 800	40.28
P_2_ 800	40.97

**Table 5:** Kinetic corrosion parameters of the OL37 carbon steel in 1 M HCl, and at different concentrations of the P_1_ inhibitor at 25 °C.

Concentration (ppm)	i_corr_ (mA·cm^−2^)	R_p_ Ω·cm^−2^	R_mpy_	P_mm/year_	K_g_ _g_/m^2^·h	−E_corr_ (mV)	b_a_ (mV·dec^−1^)	−b_c_ (mV·dec^−1^)	E (%)	θ
0	0.855	20	399	10.12	9	531	86	113	-	-
20	0.29	66	135	3.43	3.05	470	67	86	66	0.66
50	0.162	91	75.6	1.91	1.70	469	67	113	81	0.81
100	0.149	121	69.53	1.76	1.56	466	84	123	84	0.84
300	0.136	108	63.46	1.61	1.43	462	71	109	85	0.85
500	0.112	126	52.26	1.32	1.17	455	63	99	87	0.87
800	0.104	176	48.563	1.23	1.19	450	68	113	88	0.88
1000	0.059	243	25.9	0.65	0.58	488	63	81	93	0.93

**Table 6:** Corrosion kinetic parameters of the OL37 carbon steel in 1 M HCl and at different concentrations of the P_2_ inhibitor at 25 °C.

Concentration (ppm)	i_corr_ (mA·cm^−2^)	R_p_ Ω·cm^−2^	R_mpy_	P_mm/year_	K_g_ _g_/m^2^·h	−E_corr_ (mV)	b_a_ (mV·dec^−1^)	−b_c_ (mV·dec^−1^)	E (%)	θ
0	0.855	20	399	10.12	9	531	86	113	-	-
20	0.183	80	85.4	2.16	1.93	464	69	103	79	0.79
50	0.140	121	65.33	1.65	1.47	476	84	116	84	0.84
100	0.132	105	61.6	1.56	1.39	438	65	95	85	0.85
300	0.122	109	56.9	1.44	1.28	462	63	99	86	0.86
500	0.094	128	43.8	1.11	0.99	462	60	82	89	0.89
800	0.078	145	36.4	0.92	0.82	440	80	113	91	0.91
1000	0.055	236	23.33	0.59	0.53	424	56	94	94	0.94

**Table 7:** Kinetic parameters of OL37 in 1 M HCl at different immersion time of the P_1_ extract at 25 °C.

Immersion Time (h)	i_corr_ (mA·cm^−2^)	R_p_ Ω·cm^−2^	R_mpy_	P_mm/year_	K_g_ g/m^2^·h	−E_corr_ (mV)	b_a_ (mV·dec^−1^)	−b_c_ (mV·dec^−1^)	E (%)	θ
0	0.059	243	25.9	0.65	0.58	488	63	81	93	0.93
24	0.04	324	18.7	0.47	0.42	487	94	64	95	0.95
48	0.22	66	102	2.61	2.31	440	84	66	74	0.74
72	0.36	46	168	4.26	3.79	449	103	71	58	0.58
96	0.41	40	191	4.85	4.31	460	109	70	53	0.53
144	0.50	37	233	5.92	5.25	481	115	82	43	0.43

**Table 8:** Kinetic parameters of OL37 in 1 M HCl at different immersion time of the P_2_ extract at 25 °C.

Immersion Time (h)	i_corr_ (mA·cm^−2^)	R_p_ Ω·cm^−2^	R_mpy_	P_mm/year_	K_g_ g/m^2^h	−E_corr_ (mV)	b_a_ (mV·dec^−1^)	−b_c_ (mV·dec^−1^)	E (%)	θ
0	0.055	236	23.33	0.59	0.53	424	56	94	94	0.94
24	0.033	371	14.4	0.39	0.33	440	104	71	95	0.95
48	0.127	107	59.26	1.50	1.33	425	91	68	85	0.85
72	0.098	136	45.73	1.16	1.03	424	88	64	88	0.88
96	0.102	133	47.6	1.28	1.07	429	87	64	88	0.88
144	0.11	139	51.33	1.30	1.16	434	98	62	87	0.87

**Table 9:** Electrochemical–EIS parameters of the OL37 carbon steel in 1 M HCl and at different concentrations of the P_1_ inhibitor at 25 °C.

Concentration (ppm)	R_S_ ($\mathrm{ohm}\times {\mathrm{cm}}^{2}$)	Rct ($\mathrm{ohm}\times {\mathrm{cm}}^{2}$)	Q-Yo $S\cdot s^{-n}\times {\mathrm{cm}}^{-2}$	Q-n	χ
0	0.72	6.32	0.00518	0.74	4.255 × 10^−3^
20	1.00	50	0.000528	0.87	1.561 × 10^−3^
50	1.08	60	0.001102	0.89	2.525 × 10^−3^
100	0.89	60	0.000531	0.88	1.900 × 10^−3^
300	0.86	70	0.000666	0.87	1.585 × 10^−3^
500	0.79	74	0.000329	0.89	1.190 × 10^−3^
800	0.36	86	0.0004257	0.90	4.784 × 10^−3^
1000	0.78	214	0.00023	0.89	3.398 × 10^−3^

**Table 10:** Electrochemical-EIS parameters of the OL37 carbon steel in 1 M HCl and at different concentrations of the P_2_ inhibitor at 25 °C.

Concentration (ppm)	$R_{S}\;(\mathrm{ohm}\times {\mathrm{cm}}^{2})$	Rct ($\mathrm{ohm}\times {\mathrm{cm}}^{2}$)	Q-Yo $S\cdot s^{-n}\times {\mathrm{cm}}^{-2}$	Q-n	χ
0	0.72	6.32	0.00518	0.74	4.255 × 10^−3^
20	0.84	50	0.00063	0.89	1.503 × 10^−3^
50	0.72	60	0.0001653	0.88	1.814 × 10^−3^
100	0.79	67	0.000241	0.88	2.477 × 10^−3^
300	0.67	72	0.000279	0.89	1.880 × 10^−3^
500	0.69	74	0.000494	0.88	2.175 × 10^−3^
800	0.89	121	0.000196	0.89	5.169 × 10^−3^
1000	0.58	223	0.000185	0.88	3.253 × 10^−3^

**Table 11:** Electrochemical–EIS parameters of the OL37 carbon steel in 1 M HCl with 1000 ppm concentrations of the P_1_ inhibitor at different immersion times at 25 °C.

Immersion Time (h)	R_S_ ($\mathrm{ohm}\times {\mathrm{cm}}^{2}$)	Rct ($\mathrm{ohm}\times {\mathrm{cm}}^{2}$)	Q-Yo $S\cdot s^{-n}\times {\mathrm{cm}}^{-2}$	Q-n	χ
0 h	0.78	214	0.00023	0.89	3.398 × 10^−3^
24 h	0.85	152	0.00041	0.85	4.558 × 10^−3^
48 h	1.93	62	0.00529	0.82	9.875 × 10^−3^
72 h	0.63	49	0.00122	0.86	2.111 × 10^−3^
96 h	1.11	44	0.00198	0.82	1.454 × 10^−3^
144 h	1.15	38	0.002684	0.78	6.705 × 10^−3^

**Table 12:** Electrochemical-EIS parameters of the OL37 carbon steel in 1 M HCl with 1000ppm concentrations of the P_2_ inhibitor at different immersion times at 25 °C.

Immersion Time (h)	$R_{S}\;(\mathrm{ohm}\times {\mathrm{cm}}^{2})$	Rct ($\mathrm{ohm}\times {\mathrm{cm}}^{2}$)	Q-Yo $S\cdot s^{-n}\times {\mathrm{cm}}^{-2}$	Q-n	χ
0 h	0.58	223	0.00018	0.88	3.253 × 10^−3^
24 h	1.57	185	0.00851	0.88	4.570 × 10^−3^
48 h	1.91	150	0.00562	0.87	2.178 × 10^−3^
72 h	1.22	179	0.00193	092	1.640 × 10^−3^
96 h	1.85	166	0.00145	0.89	2.631 × 10^−3^
144 h	1.28	118	0.00463	0.86	2.983 × 10^−3^

**Table 13:** The values of K_ads_ and $∆G_{ads}^{{}^{\circ}}$ for the P_1_ and P_2_ extracts on OL37 in 1 M HCl.

The System	K_ads_, M^−1^	$∆G_{ads}^{{}^{\circ}}$ (KJmol^−1^)	The Type of Adsorption
P_1_	2.17 × 10^4^	−34.09	Chemisorption and physical adsorption
P_2_	3.91 × 10^4^	−35.51	Chemisorption and physical adsorption

**Table 14:** OL37 elemental composition.

C (%)	Si (%)	Mn (%)	Fe (%)	P (%)	S (%)	Al (%)	Ni (%)	Cr (%)
0.15	0.09	0.4	99.293	0.023	0.02	0.022	0.001	0.001

## Data Availability

The original contributions presented in this study are included in the article/Supplementary Materials. Further inquiries can be directed to the corresponding authors.

## Supplementary Materials

The following supporting information can be downloaded at: https://www.mdpi.com/article/10.3390/molecules30122660/s1.

## Abbreviations

The following abbreviations are used in this manuscript:

RTLE	*Rhus typhina* L. leaves extract
HPLC-DAD	High-performance liquid chromatography with diode-array detection
LC-MS	Liquid chromatography–mass spectrometry
SEM	Scanning electron microscope
EDX	Energy dispersive X-ray analysis
GCI	Green corrosion inhibitors
OCP	Open circuit potential
EIS	Electrochemical impedance spectroscopy
PDP	Potentiodynamic polarization
XPS	X-ray photoelectron spectroscopy
AFM	Atomic force microscopy
RefE	reference electrode
WE	working electrode OL7
CE	platinum sheet counter electrode.
