# Supplementary material for: Electrochemical Assessment of Rhus typhina L. Leaf Extract as a Novel Green Corrosion Inhibitor for OL37 in 1 M HCl Medium
Source: Molecules. 2025 Jun 19;30(12):2660. doi: 10.3390/molecules30122660 (PMC12196263; doi:10.3390/molecules30122660)

04/14/23 04:44:25

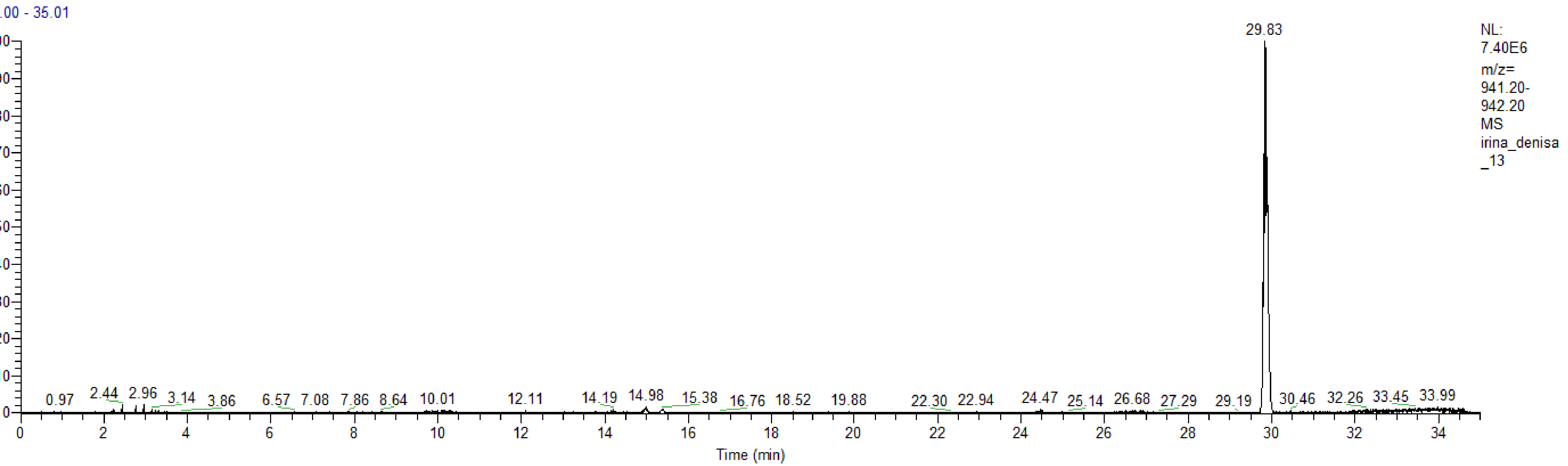

denisa_13 #6698 RT: 29.85 AV: 1 NL: 5.28E7
MS + p ESI Full ms [100.00-1000.00]

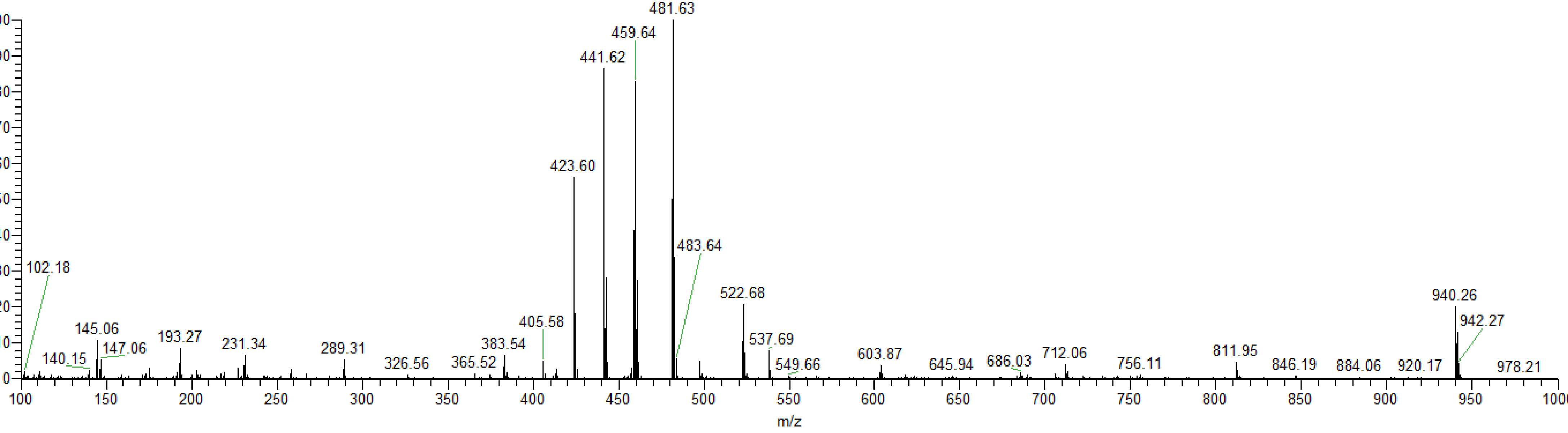

04/14/25 05:56:23

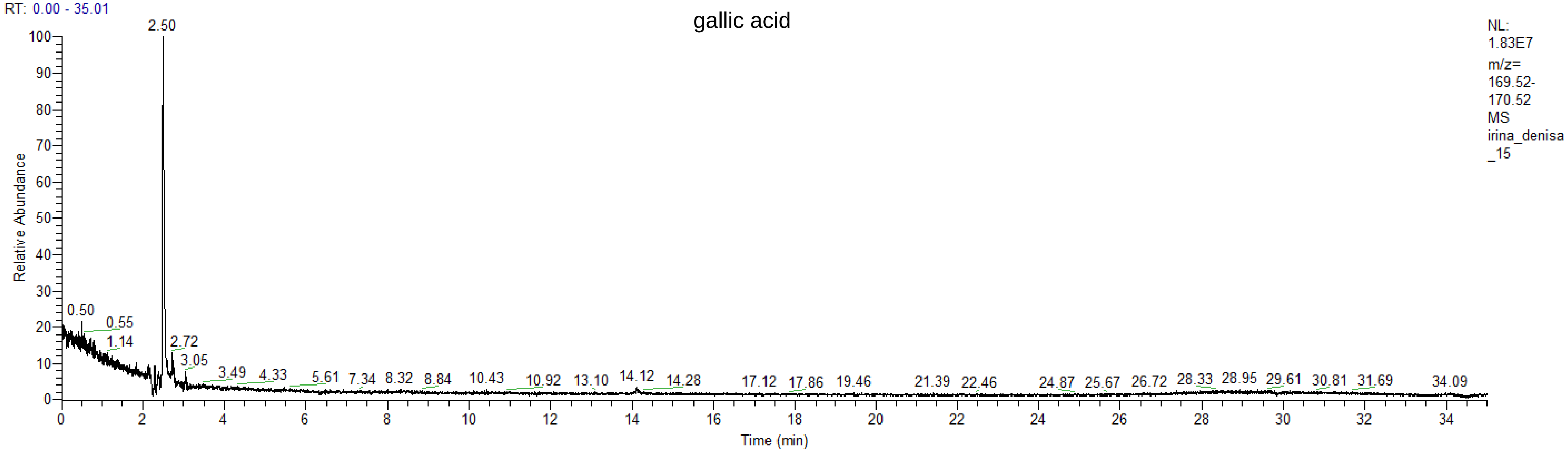

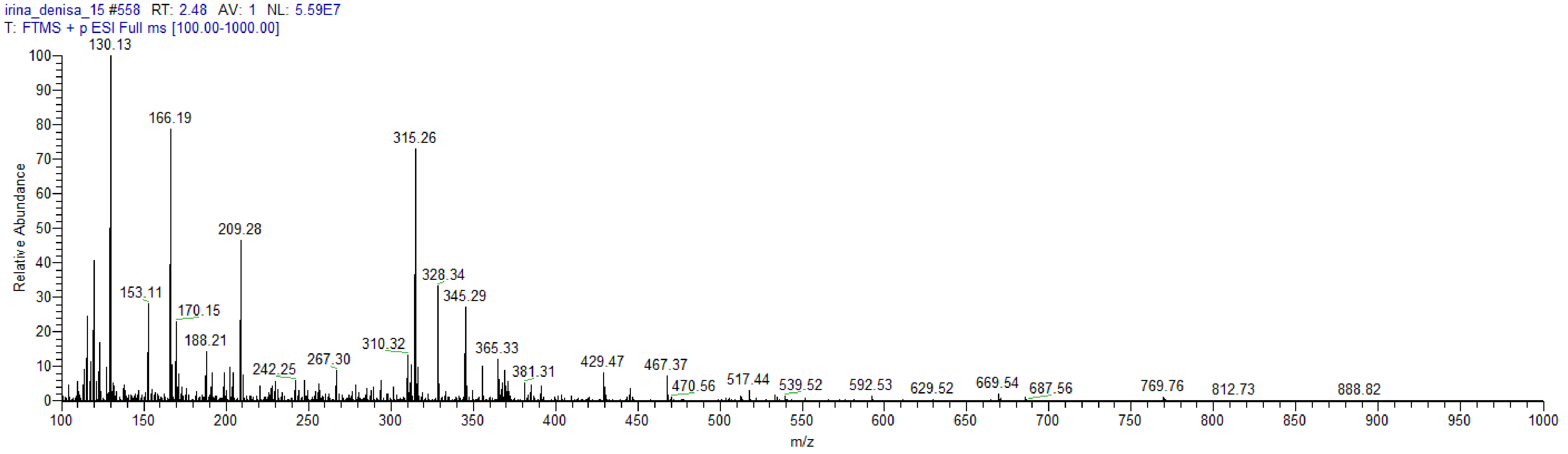

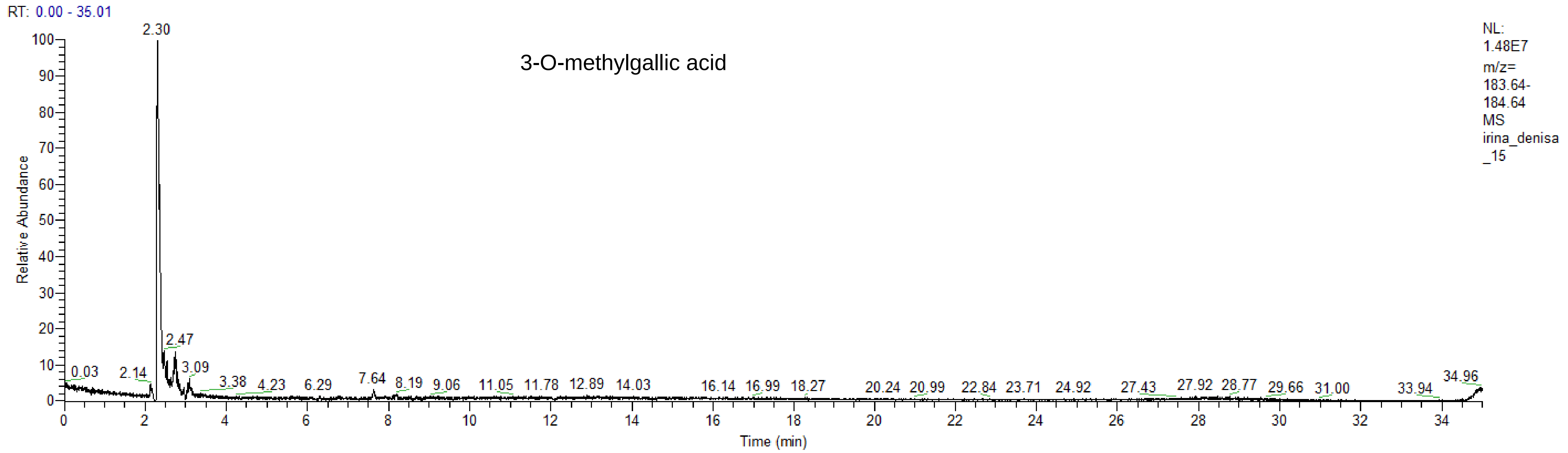
RT: 0.00 - 35.01
3-O-methylgallic acid
NL: 1.48E7 m/z= 183.64-184.64 MS irina_denisa_15
2.30
2.47
3.09
0.03
2.14
3.38
4.23
6.29
7.64
8.19
9.06
11.05
11.78
12.89
14.03
16.14
16.99
18.27
20.24
20.99
22.84
23.71
24.92
27.43
27.92
28.77
29.66
31.00
33.94
34.96
Relative Abundance
Time (min)

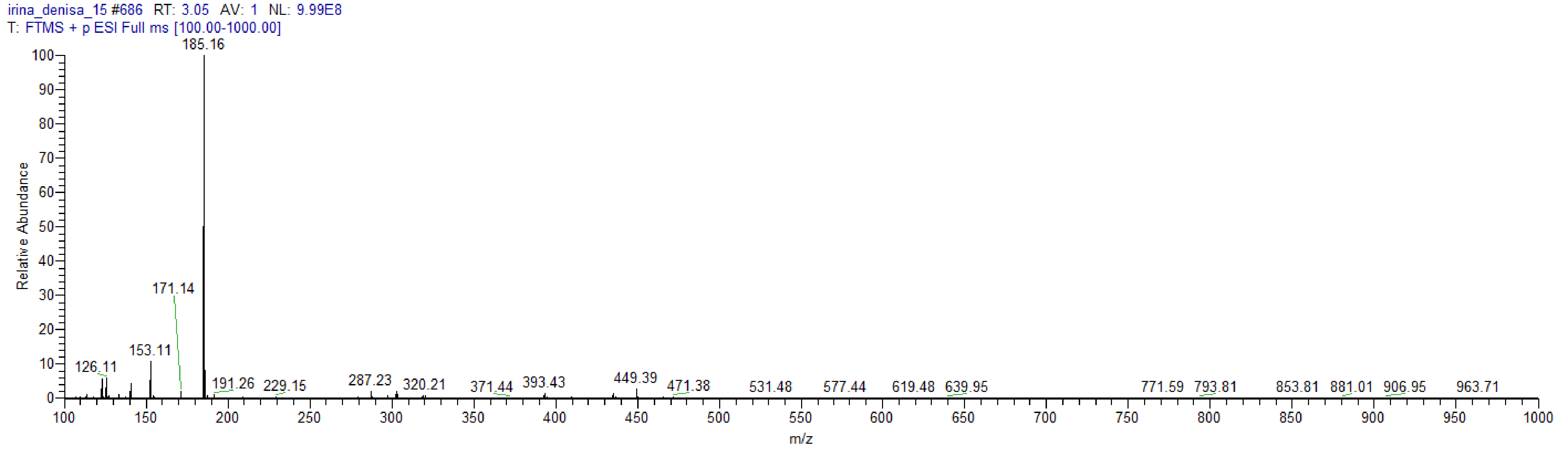
irina_denisa_15 #686 RT: 3.05 AV: 1 NL: 9.99E8
T: FTMS + p ESI Full ms [100.00-1000.00]
185.16
171.14
153.11
126.11
191.26
229.15
287.23
320.21
371.44
393.43
449.39
471.38
531.48
577.44
619.48
639.95
771.59
793.81
853.81
881.01
906.95
963.71
Relative Abundance
m/z

Chromatograms for used and not used extracts at low concentrations

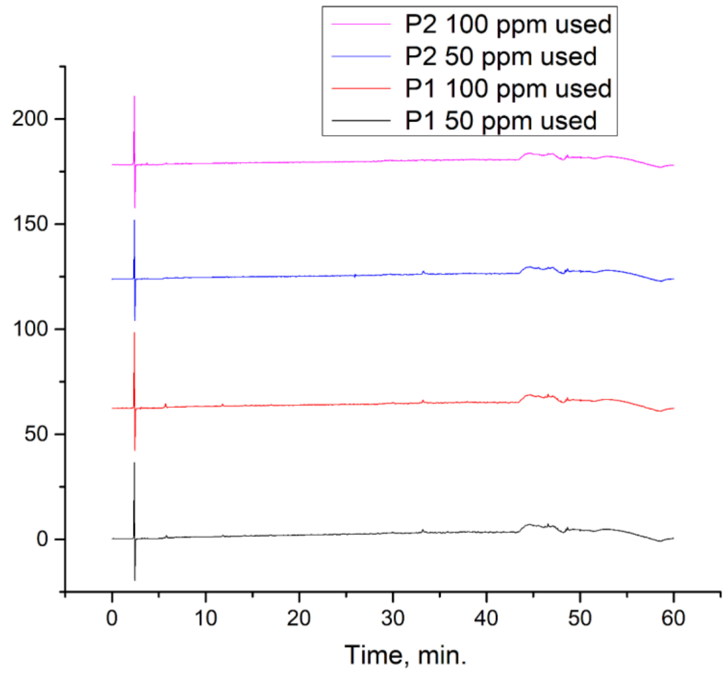

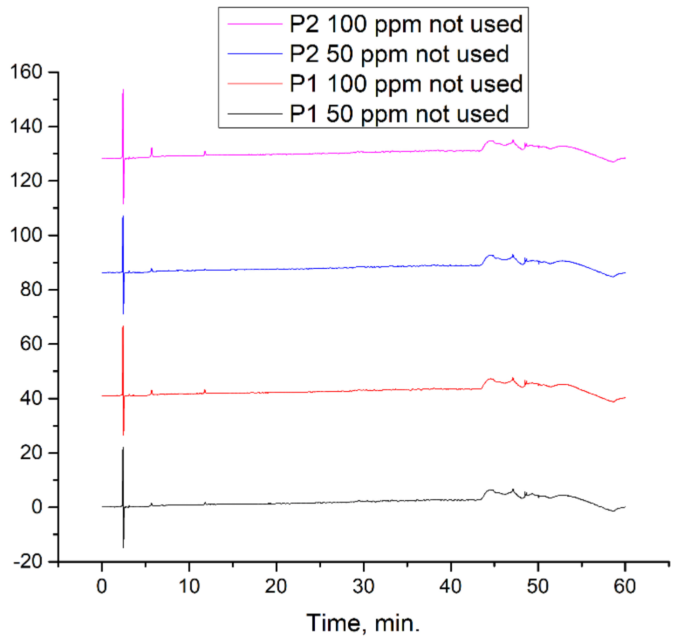

Supplement: Supplementary file 1 [file molecules-30-02660-s001.zip › molecules-3667263-supplementary.pdf]
